# ABO incompatibility and component irradiation are independently associated with platelet transfusion reaction rate

**DOI:** 10.1111/trf.18130

**Published:** 2025-01-13

**Authors:** Keenan O. Hogan, Geethanjalee Mudunkotuwa, Milind Phadnis, X. Long Zheng, Zhan Ye

**Affiliations:** 1 Department of Pathology and Laboratory Medicine, The University of Kansas Medical Center, Kansas City, Kansas, USA; 2 Department of Biostatistics and Data Science, The University of Kansas Medical Center, Kansas City, Kansas, USA; 3 Institute of Reproductive Medicine and Developmental Sciences, The University of Kansas Medical Center, Kansas City, Kansas, USA

**Keywords:** compatibility, irradiation, platelet, reaction, transfusion

## Abstract

**Background:**

Allocating incompatible platelet components to avoid product wastage must be balanced against the risk of reduced efficacy and adverse outcomes. The impact of platelet compatibility in association with product irradiation or pathogen reduction is unknown. This study aims to determine the combined and independent impact of platelet compatibility and component modification on transfusion reaction rate.

**Study Design and Methods:**

A retrospective review of all adult platelet transfusions from 2020 to 2022 was performed, including all reported reactions. Logistic regression was performed to evaluate the significance of ABO compatibility and unit modification for reaction rate.

**Results:**

Out of 21,330 transfusions to 3450 patients, 285 (1.33%) reactions were reported and 178 (0.83%) were diagnosed as related to transfusion, predominantly febrile nonhemolytic (*n* = 59) and allergic (*n* = 102). The compatibility of transfusion was 67.7% ABO identical, 13.8% ABO minor incompatible, 17.2% ABO major incompatible, and 1.4% ABO bidirectionally incompatible. Irradiated, unmodified, and pathogen-reduced single-donor platelets were transfused in 70.9%, 21.8%, and 7.3% of cases, respectively. Univariable regression demonstrated increased odds of reaction for major incompatibility vs. ABO identical (OR: 1.92; 95% CI: 1.36–2.71) and irradiated vs. unmodified (OR: 2.34; 95% CI: 1.45–3.91), which were confirmed by multivariable analysis. The effect of compatibility and unit modification were independent in all analyses.

**Conclusions:**

The results demonstrate a trend of increasing reaction rate associated with major incompatibility and product irradiation. This study provides additional data to inform institutional policies guiding product selection for individual patients.

## 1 | INTRODUCTION

Leukoreduced apheresis/single-donor platelets (SDPs) constitute the majority of platelet transfusions in the United States (~60%) with a high rate of irradiation (~70%) due to demand driven by chemotherapy-induced thrombocytopenia in patients with hematologic neoplasms.^1^ The presence of A and B antigens on platelets combined with the relatively large amount of plasma present in SDPs presents a dual concern for antigen and antibody ABO incompatibility for each transfusion. Platelet transfusions can be characterized as ABO identical/compatible, plasma/minor incompatible (e.g., O donor to A patient), platelet/major incompatible (e.g., A donor to O patient), and dually/bidirectionally incompatible (e.g., A donor to B patient). The reported rates for ABO identical (~50%), plasma incompatible (~20%), and platelet incompatible (~30%) transfusions have been attributed to inventory management optimized to avoid waste.^1,2^ However, prioritization of waste reduction must be considered in the context of clinical outcomes associated with the compatibility of transfused platelets, including reduced post-transfusion platelet increment, increased platelet refractoriness, hemolysis, and even increased mortality for certain subgroups.^3–6^

The risk of transfusion reaction following platelet transfusion is generally well-recognized and considered by clinicians and institutional transfusion services. However, evidence regarding the impact of platelet compatibility on the rate of transfusion reaction is unclear. Two separate studies with different designs have reported contradictory conclusions on the significance of platelet compatibility to transfusion reaction rate.^7,8^ The more recent study by Malvik et al., a large retrospective single-center review, showed a significant increase in total reported and transfusion-related reactions in the combined ABO-incompatible group and major-incompatible subgroup.^8^ Both studies concluded platelet storage duration had no significant impact on transfusion reaction rate, but neither study included analysis of the combined impact of compatibility with the potential confounder of unit modification: pathogen-reduced (prSDP) or gamma-irradiated (iSDP) compared with unmodified leukoreduced units (nSDP).

While previous studies did not show a consistently significant difference between iSDP, prSDP, and nSDP, transfusion of prSDP was reported to be associated with an overall decrease in adverse reactions, particularly allergic reactions.^9–14^ To our knowledge, concurrent comparison of platelet compatibility and unit modification regarding transfusion reaction rate has not been reported. The aim of this study is to determine the combined and independent impact of platelet compatibility and unit modification on transfusion reaction rate.

## 2 | STUDY DESIGN AND METHODS

This single-center retrospective study was approved by our institutional review board (IRB#00000161), which provides oversight for the academic medical center, including a hematopoietic stem cell transplant center and level 1 trauma center. Patient and transfusion data from January 2020 to December 2022 were exported from the laboratory information system (Sunquest Information Systems, Arizona, USA) to include all complete and partial platelet transfusions for adult patients. All units transfused were leukoreduced SDPs classified as large-volume delayed sampling, with or without irradiation (JL Shepherd, model 143-512, cesium-137, California, USA), or pathogen-reduced (INTERCEPT, Cerus Co., California, USA). Most platelets are irradiated at receipt into inventory due to an expected high iSDP transfusion volume; however, additional units are often irradiated after receipt and up to immediately prior to transfusion due to receipt of HLA-matched units and unexpected spikes in transfusion requests on a given day. This study did not track unit age at the time of irradiation or transfusion. Platelet products at our institution are not routinely stored using platelet additive solution (PAS). Although this was not specifically tracked in the current study, we would anticipate transfusion of a unit stored in PAS to be a rare event.

Exclusion criteria for transfusion included when the unit was washed or when a definitive ABO type within 3 days prior to transfusion was not available (secondary to hematopoietic stem cell transplant chimerism or emergency release prior to ABO typing). Patient age was determined by the number of full years between date of birth and date of transfusion. Unit compatibility was determined from direct comparison of proximal pre-transfusion patient ABO type and unit ABO type for each transfusion. Platelet ABO compatibility was classified as identical (ABOid) or ABO incompatible (ABOin) due to plasma/minor incompatibility (ABOmin), platelet/major incompatibility (ABOmaj), or dual/bidirectional incompatibility (ABObi) as depicted in Table 1.

Paper copies of the laboratory evaluation, including clerical checks, visual plasma inspection, repeat ABO-Rh testing, direct antiglobulin testing, urine hemoglobin/microscopy, and clinical investigation for all reported suspected transfusion reactions, were evaluated. Transfusion reaction classification followed Centers for Disease Control and Prevention National Healthcare Safety Network (NHSN) hemovigilance guidelines to include diagnosis for all reported cases, and severity and imputability for all cases diagnosed as related to transfusion.^15^ Reaction data was matched to transfusion data through unique platelet unit identification number cross-checked by patient name and date/time of transfusion.

The estimated sample size was determined based on a power of 80%, a baseline reported transfusion reaction rate of 1%, and an expected ratio of 2:1 ABOid:ABOin. To detect a difference of 0.5% across 12 pairwise comparisons, we established a minimum of ~13,500 transfusions to be evaluated. To enhance the sensitivity of the analysis, we included all cases starting on the date when transfusion reaction evaluation hard copies were available on site. The expected proportional transfusion reaction rate for each subgroup was based on platelet transfusion distribution across two variables: platelet unit type (nSDP, iSDP, prSDP) and compatibility (ABOid, ABOmin, ABOmaj, ABObi). Pre-specified combinations of ABOid/ABOmin, ABOmaj/ABObi, and nSDP/prSDP were also evaluated to account for low-incidence subgroups. Repeat analysis was performed for reactions diagnosed as related to transfusion with a definite or probable imputability, allergic reactions alone, and febrile nonhemolytic transfusion reactions alone.

Descriptive statistics were generated for transfusion characteristics. The continuous variable of age was reported using mean and standard deviation. All other characteristics were reported with frequency counts and proportions. Treating each transfusion as an independent event, a univariable logistic regression analysis was conducted to assess if platelet compatibility or unit modification was associated independently with the occurrence of transfusion reactions. Additionally, multiple logistic regression was done to assess the combined effect of compatibility and unit modification adjusted for age. These models were also evaluated for the presence of an interaction between compatibility and unit modification. All pairwise comparisons were adjusted for multiplicity using Holm’s method. An additional sensitivity analysis was performed using a logistic regression with random intercept for each subject to evaluate for bias introduced by individual patients experiencing multiple transfusions. We also obtained an aggregate summary of transfusion reactions cross tabulated by compatibility and unit modification. This allowed us to perform a Poisson regression analysis modeling the impact of compatibility and unit modification on transfusion reaction rates. All data analyses were conducted using the R and SAS statistical software and statistical significance was set at the 5% level.

## 3 | RESULTS

A total of 3487 adult patients received 21,512 SDP transfusions during the study period. Following exclusion of washed platelets (3 patients; 11 transfusions) and patients without definitive ABO type within 3 days of transfusion (34 patients; 171 transfusions), there were 3450 unique patients and 21,330 platelet transfusions analyzed. Patients’ mean age was 56.3 years (SD = 15.7 years) with a range of 18–97 years. Among analyzed transfusions, 9745 (45.7%) were for patients with blood type O, 8291 (38.9%) type A, 2558 (12%) type B, and 736 (3.5%) type AB. Compatibility was 67.6% ABOid, 17.2% ABOmaj, 13.8% ABOmin, and 1.4% ABObi. Unit modification included irradiation for 70.9% and pathogen reduction for 7.3%, with no modification in the remaining 21.8% (Table 2). This table also provides a breakdown of these baseline characteristics for all transfusions without reported reactions, with reported reactions, and with diagnosed transfusion-associated reactions, including comparison by independent two-sample *t*-test and *Z*-test of proportions. Significance was achieved for a marginal difference in age with younger patients more likely to experience a suspected reaction and a reaction related to transfusion. Reported reactions and reactions related to transfusion were common for ABOmaj transfusions and less common for ABOid transfusions. Similarly, a reaction was reported and diagnosed as related to transfusion more frequently when irradiated products were used and less frequently when an unmodified product was transfused.

Transfusions excluded from analysis due to the absence of a definitive ABO type were associated with two reported reactions, leaving 285 reported suspected reactions (1.33% of total transfusions), 178 of which were diagnosed as being related to a single platelet transfusion by a Transfusion Medicine physician according to NHSN guidelines (0.83% of total transfusions): acute hemolytic transfusion reaction (AHTR = 1), allergic transfusion reaction (ATR = 102), febrile nonhemolytic transfusion reaction (FNHTR = 59), hypotensive transfusion reaction (HyTR = 4), transfusion-associated circulatory overload (TACO = 8), transfusion-related acute lung injury (TRALI = 1), and transfusion-transmitted infection (TTI = 3) (Table 3). This table also provides the transfusion reaction evaluation determination by diagnostic category across transfusion compatibility and product modification. Imputability was probable or definite for 151 reactions (0.71% of total transfusions). Twelve reactions were graded as severe: 1/1 TRALI, 2/8 TACO, and 9/102 ATR. Seven of the severe ATR were associated with ABOin units. Irradiated units were associated with 3/3 TTI and 4/4 HyTR. The single AHTR was non-severe and occurred with an O-Rh negative iSDP transfused to an A-Rh positive patient.

**FIGURE 1:**
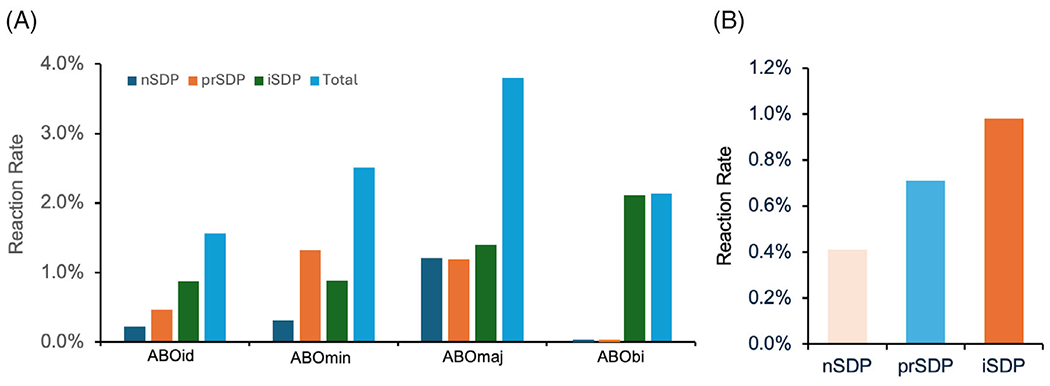
Proportional reaction rates. (A) Subgroup and total reaction rates are grouped by compatibility. (B) Total reaction rates grouped by unit modification. ABObi, ABO bidirectionally incompatible; ABOid, ABO identical; ABOmaj, ABO major incompatible; ABOmin, ABO minor incompatible; iSDP, gamma irradiated; nSDP, unmodified; prSDP, pathogen reduced.

A comparison of proportional transfusion-related reaction rate across unit compatibility and modification is demonstrated in Table 4 and Figure 1. The overall trend of increased reactions with ABOmaj and, to a lesser extent, with ABOmin holds true for nSDP and iSDP. Transfusing ABOmin compared to ABOid made a minor to marginal difference for nSDP (0.22% vs. 0.31%) and iSDP (0.87% vs. 0.88%). For prSDP, the relative rate of reaction is increased for ABOmin (1.32%) compared to both ABOid (0.47%) and ABOmaj (1.19%), although the overall incidence is low due to the small number of prSDP transfused. Within each compatibility group, the overall trend of increased reactions with prSDP and iSDP is only reflective of ABOid reactions. Among ABOmaj transfusions, there is only a marginal difference between nSDP (1.21%) and prSDP (1.19%) reaction rate. When considering all reported reactions, the proportional reaction rates increase slightly across all subcategories but maintain the same ordinal positions relative to one another (data not shown).

Univariable and multivariable logistic regressions were conducted to compare the odds of having a reaction by unit compatibility and modification subtypes. As shown in Table 5, statistical significance was reached when comparing the odds ratio (OR) of having reactions related to transfusion with ABOmaj versus ABOid (OR: 1.92; 95% CI: 1.36–2.71), ABOmaj versus ABOmin (OR: 1.72; 95% CI: 1.05–2.83), iSDP versus nSDP (OR: 2.34; 95% CI: 1.45–3.91), ABOmaj/ABObi versus ABOid/ABOmin (OR: 1.93; 95% CI: 1.36–2.71), and iSDP versus nSDP/prSDP (OR: 2.24; 95% CI: 1.50–3.78). The same subgroup comparisons redemonstrated statistical significance in a multivariable analysis. There was no statistical evidence of an interaction between compatibility and unit modification in either analysis. Unit and patient ABO varied by compatibility category, as expected. Repeat analysis including only those reactions diagnosed with an imputability of definite or probable; only ATR diagnoses; and only FNHTR diagnoses showed similar proportional reaction rates and statistically significant group comparisons (data not shown).

Sensitivity analysis adjusted for individual patients experiencing multiple transfusions demonstrated no significant change in primary or subgroup comparisons, supporting statistical interpretation of each transfusion as an independent event (data not shown). A final exploratory sensitivity analysis was performed using Poisson regression. Modeling at the aggregate level, the predicted rates of a reported reaction based on unit modification and compatibility group redemonstrated the trends of the primary analyses (Table 6). The highest model-based predicted probability of a transfusion reaction was for patients receiving an iSDP unit that was ABOmaj (2.9%) or ABObi (3.6%).

## 4 | DISCUSSION

The clinical significance of platelet transfusion compatibility has evolved in recent years. Historically, the focus of laboratory policy centered on the avoidance of ABOmin transfusions due to the risk of potentially life-threatening hemolysis. Due to the logistical challenge of maximizing ABOid transfusions without significantly increasing inventory waste, transfusion services avoiding ABOmin see increased transfusion of ABOmaj. As both platelet count increment and time to next transfusion decrease with ABOmaj transfusions, the loss of inventory is only partially mitigated.

Early transfusion cessation due to a suspected transfusion reaction also risks the need for subsequent transfusion, compounding inventory waste. Our data supports the results of the study by Malvik et al.,^8^ which demonstrated a statistically significant increase in suspected and diagnosed transfusion reactions with ABOmaj transfusions. Combined, these two single-institution retrospective reviews include nearly 6000 unique patients receiving over 34,000 platelet transfusions associated with over 450 reported reactions. We also tested the more conservative measure of transfusion reactions diagnosed with high imputability and found the associations with unit compatibility were maintained. The current study additionally provides evidence that product irradiation is independently associated with increased transfusion reaction rate.

The additive impact of product modification and unit compatibility on the reaction rate was not proportional (e.g., the empirical risk for nSDP/ABOmaj (1.21%) was greater than iSDP/ABOid (0.88%) and likely represents different etiologies). The increase in reactions following ABOmaj transfusions, particularly the more common ATR and FNHTR, may be ascribed to immunomodulation secondary to circulating immune complexes.^16,17^ To our knowledge, the mechanism has not been described, but may be reasonably proposed to involve immune complex stimulation of dendritic cells and complement mediators, leading to downstream chemotaxis, cytokine signaling, reactive oxygen species production, and mast cell histamine release. Distinguishing which factors may determine the development or suppression of a febrile vs. an allergic response to these stimuli requires additional study. Transfusion of inflammatory mediators generated during component preparation and storage may also play a role, although storage duration has not been shown to significantly affect reaction rate.^7,8^ However, this relationship may be confounded by timing of irradiation, which is not reported in the current or previous studies and may blur the timeframe for natural development of platelet storage lesions by introducing increased oxidative stress.^18,19^

In addition to the possible role of radiation-induced oxidative stress of platelet components, we suspect that the increase in reactions associated with iSDP most likely represents a combination of factors common among patients requiring product irradiation, who routinely undergo conditioning and consolidative chemotherapy around hematopoietic stem cell transplant. This patient population often suffers from iatrogenic pancytopenia, necessitating multiple transfusions of platelet and red blood cell concentrates, as well as complex immune dysregulation. These conditions may increase patient sensitivity to infusion and/or stimulation of inflammatory mediators, lowering the threshold for the development of a febrile or allergic response. Repeat random donor exposures may also broaden IgG-mediated allergic response potential even as allergen-specific IgE responses decline with chemotherapy.^20,21^ Immune complex formation associated with transfusion has also been shown to impact hemostasis through inhibition of platelet function and clot formation kinetics, in addition to the well-characterized platelet refractoriness associated with ABOmaj transfusion.^17^ Indeed, a retrospective analysis by Bougie et al.,^6^ in 2023, linked increased mortality in hematology/oncology patients with blood groups A and B (along with intracerebral hemorrhage patients with blood group O) with ABOmaj platelet transfusions. While the current study did not assess for mortality, it is likely that similar underlying mechanisms contribute to the association between major incompatible transfusions of irradiated platelets and the rate of reaction in this patient population.

This single-institution retrospective analysis, although the largest to date on this topic, appears to still be underpowered for detecting statistically significant differences in certain subgroup combinations. The confidence interval for ABObi suggests that a larger sampling may demonstrate a statistically significant effect on the odds of reaction. However, our dataset also demonstrates a trend toward an increased rate of product irradiation for ABObi transfusions that may confound even a larger sampling. We attribute this trend to two primary clinical situations at our institution: ABO status being a secondary consideration for the selection of HLA-matched platelets and transfusing to protect engraftment when AB platelets are not available. Another anomaly that cannot be fully explained is the relatively increased rate of reaction for prSDP/ABOmin (1.32%). Pathogen reduced units only represented 7.3% of transfusions and the three reactions in this subgroup consisted proportionally of more ATR (2:1) than nSDP/ABOmin (1:1) and iSDP/ABOmin (1.25:1). These associations may be confounded by infrequent use of PAS in prSDP at our institution as PAS replacement of donor plasma has been shown to mitigate adverse reactions.^22^ It may require a significantly larger multicenter dataset accounting for additional variables to further explore these subgroup comparisons, although this would introduce additional difficulties of accounting for variation in diagnostic evaluation and product selection policies.

In summary, this study provides further evidence that ABOmaj platelet transfusions are 1.5–2.0 times more likely to be associated with a transfusion reaction, even when excluding reactions diagnosed with low imputability. Platelet irradiation was shown to be independently associated with a 2.0–2.5 times increase in the likelihood of a transfusion reaction. The impact of these two factors was additive but not proportional. Further research may expand on these associations to support their use as an adjunct consideration in choosing product compatibility, given individual patient product needs. Our institution is reexamining the current unit compatibility decision tree for certain patient subpopulations in the context of these findings. A future prospective evaluation of a modified institutional product selection guide would provide additional evidence on logistical viability and association with additional patient outcomes.

## Figures and Tables

**TABLE 1:** Platelet transfusion compatibility determination by patient and donor ABO status.

	Patients			
	AB	A	B	O
Donor				
AB	ABOid	ABOmaj	ABOmaj	ABOmaj
A	ABOmin	ABOid	ABObi	ABOmaj
B	ABOmin	ABObi	ABOid	ABOmaj
O	ABOmin	ABOmin	ABOmin	ABOid

Abbreviations: ABObi, ABO bidirectionally incompatible (transfusion of incompatible antigen and antibody); ABOid, ABO identical (transfusion of compatible antigen and antibody); ABOmaj, ABO major incompatible (transfusion of incompatible antigen); ABOmin, ABO minor incompatible (transfusion of incompatible antibody).

**TABLE 2:** Transfusion characteristics by reaction group.

	No reaction (*n* = 21 045)	Reported reaction (*n* = 285)	Diagnosed reaction (*n* = 178)	Total (*n* = 21 330)	*p*-Value (NR vs. RR)	*p*-Value (NR vs. DR)
Patient age^a^						
Mean (SD)	56.3 (15.7)	53.7 (16.7)	53.1 (17.2)	56.3 (15.7)	.0094	.0143

Patient ABO^b^						
O	9605 (45.6%)	140 (49.1%)	94 (52.8%)	9745 (45.7%)	.2659	.0663
A	8189 (38.9%)	102 (35.8%)	58 (32.6%)	8291 (38.9%)	.3111	.0995
B	2528 (12.0%)	30 (10.5%)	16 (9.0%)	2558 (12.0%)	.4995	.2623
AB	723 (3.4%)	13 (4.6%)	10 (5.6%)	736 (3.5%)	.3837	.1670

Compatibility^b^						
ABOid	14,253 (67.7%)	174 (61.1%)	101 (56.7%)	14,427 (67.6%)	.0199	.0024
ABOmin	2906 (13.8%)	38 (13.3%)	23 (12.9%)	2944 (13.8%)	.8851	.8161
ABOmaj	3597 (17.1%)	65 (22.8%)	49 (27.5%)	3662 (17.2%)	.0138	.0004
ABObi	289 (1.4%)	8 (2.8%)	5 (2.8%)	297 (1.4%)	.0648	0.1020

Unit modification^b^						
iSDP	14,876 (70.7%)	243 (85.3%)	148 (83.1%)	15,119 (70.9%)	<.0001	.0004
prSDP	1541 (7.3%)	15 (5.3%)	11 (6.2%)	1556 (7.3%)	.2251	.6610
nSDP	4628 (22.0%)	27 (9.5%)	19 (10.7%)	4655 (21.8%)	<.0001	.0004

Abbreviations: ABObi, ABO bidirectionally incompatible; ABOid, ABO identical; ABOmaj, ABO major incompatible; ABOmin, ABO minor incompatible; DR, reaction related to transfusion diagnosed; iSDP, gamma irradiated; NR, no reaction reported; nSDP, unmodified; prSDP, pathogen reduced; RR, suspected reaction reported for further evaluation.

a Significance determined by two-sample *t*-test.

b Significance determined by two-sample proportion test.

**TABLE 3:** Transfusion reaction category by transfusion compatibility and unit modification.

	Compatibility				Unit modification			Total
	ABOid	ABOmin	ABOmaj	ABObi	nSDP	prSDP	iSDP	
Diagnosis								
Transfusion unrelated	73 (42%)	15 (39.5%)	16 (24.6%)	3 (37.5%)	8 (29.6%)	4 (26.7%)	95 (39.1%)	107 (37.5%)
ATR	51 (29.3%)	13 (34.2%)	34 (52.3%)	4 (50%)	11 (40.7%)	7 (46.7%)	84 (34.6%)	102 (35.8%)
FNHTR	39 (22.4%)	9 (23.7%)	10 (15.4%)	1 (12.5%)	5 (18.5%)	3 (20.0%)	51 (21.0%)	59 (20.7%)
TACO	6 (3.4%)	0	2 (3.1%)	0	2 (7.4%)	1 (6.7%)	5 (2.1%)	8 (2.8%)
TRALI	1 (0.6%)	0	0	0	1 (3.7%)	0	0	1 (0.3%)
HyTR	2 (1.1%)	0	2 (3.1%)	0	0	0	4 (1.6%)	4 (1.4%)
TTI	2 (1.1%)	0	1 (1.5%)	0	0	0	3 (1.2%)	3 (1.1%)
AHTR	0	1 (2.6%)	0	0	0	0	1 (0.4%)	1 (0.3%)

Total	174	38	65	8	27	15	243	285

Abbreviations: ABObi, ABO bidirectionally incompatible; ABOid, ABO identical; ABOmaj, ABO major incompatible; ABOmin, ABO minor incompatible; AHTR, acute hemolytic transfusion reaction; ATR, allergic transfusion reaction; FNHTR, febrile nonhemolytic transfusion reaction; HyTR, hypotensive transfusion reaction; iSDP, gamma irradiated; nSDP, unmodified; prSDP, pathogen reduced; TACO, transfusion-associated circulatory overload; TRALI, transfusion-related acute lung injury; TTI, transfusion-transmitted infection.

**TABLE 4:** Reactions related to transfusion as a proportion of total transfusions.

	Compatibility				
	ABOid	ABOmin	ABOmaj	ABObi	Total
Unit modification					
nSDP	7/3138 (0.22%)	2/649 (0.31%)	10/826 (1.21%)	0/42 (0%)	19/4655 (0.41%)
prSDP	5/1059 (0.47%)	3/228 (1.32%)	3/253 (1.19%)	0/16 (0%)	11/1556 (0.71%)
iSDP	89/10,230 (0.87%)	18/2067 (0.88%)	36/2583 (1.40%)	5/239 (2.11%)	148/15,119 (0.98%)

Total	101/14,427 (0.70%)	23/2944 (0.78%)	49/3662 (1.34%)	5/297 (1.68%)	178/21,330 (0.83%)

Abbreviations: ABObi, ABO bidirectionally incompatible; ABOid, ABO identical; ABOmaj, ABO major incompatible; ABOmin, ABO minor incompatible; iSDP, gamma irradiated; nSDP, unmodified; prSDP, pathogen reduced.

**TABLE 5:** Logistic regression analysis of reactions related to transfusion.

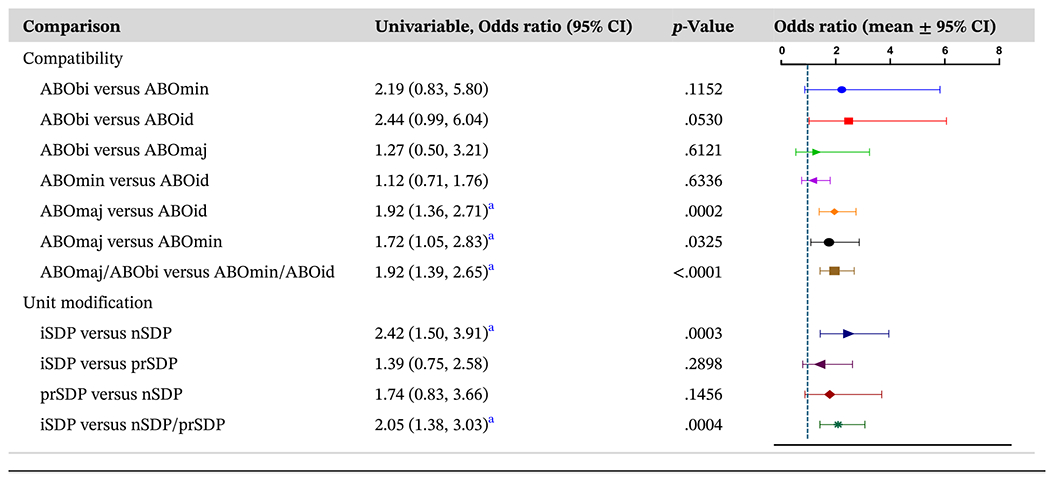

Abbreviations: ABObi, ABO bidirectionally incompatible; ABOid, ABO identical; ABOmaj, ABO major incompatible; ABOmin, ABO minor incompatible: iSDP, gamma irradiated; nSDP, unmodified; prSDP, pathogen reduced.

a Maintained significance in multiple logistic regression analysis, including adjustment for multiple transfusions per patient; dashed vertical line indicates odds ratio of 1.

**TABLE 6:** Predicted reaction rates by Poisson regression modeling.

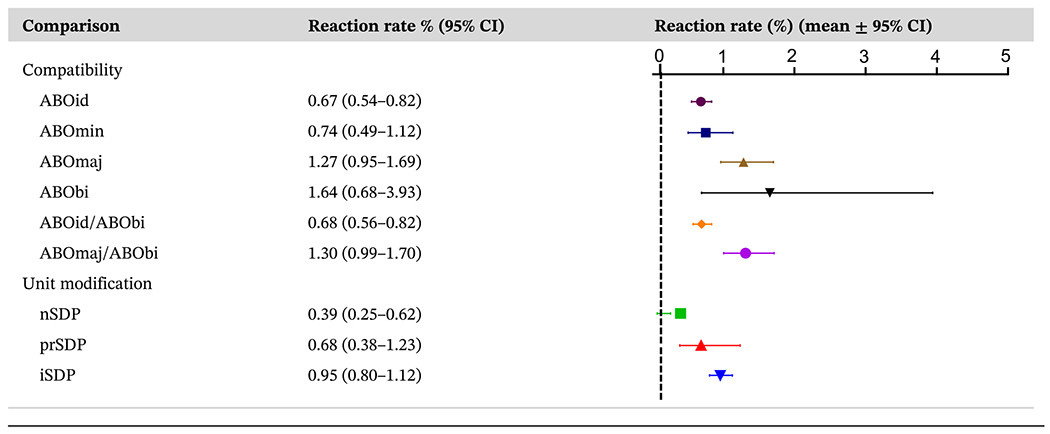

Abbreviations: ABObi, ABO bidirectionally incompatible; ABOid, ABO identical; ABOmaj, ABO major incompatible; ABOmin, ABO minor incompatible; iSDP, gamma irradiated; nSDP, unmodified; prSDP, pathogen reduced.

## Abbreviations:

ABObi: dual/bidirectional incompatibility
ABOid: platelet ABO identical
ABOin: ABO incompatible
ABOmaj: platelet/major incompatibility
ABOmin: plasma/minor incompatibility
AHTR: acute hemolytic transfusion reaction
ATR: allergic transfusion reaction
FNHTR: febrile nonhemolytic transfusion reaction
HLA: human leukocyte antigen
HyTR: hypotesive transusion reaction
iSDP: irradiated SDP
nSDP: unmodified units of SDP
prSDP: pathogen-reduced SDP
PSA: platelet additive soluton
SDPs: leukoreduced apheresis/single-donor platelets
TACO: transfusio-associated circulatory overload
TRALI: transfusion-related acute lung injury
TTI: transfusion-transmitted infection
